# Appendix to the Report of the London Committee of Apothecaries and Surgeon-Apothecaries

**Published:** 1813-12

**Authors:** 


*For the Medical and Physical Journal.*

APPENDIX *to the* REPORT *of the* LONDON COMMITTEE *of* APOTHECARIES *and* SURGEON-APOTHECARIES.

(*Continued from p.* 392.)

No. VIII.

SIR, *Brook Street, Jan.* 2, 1813.

THE ordinary Meeting of the College will be on the 12th of next April; but finding that it is the wish of the Committee of Apothecaries that their Report should be submitted to to the College of Physicians at an early period, I will take the first convenient opportunity (which I hope will occur in this month) to lay it before them.

I have the honor to be, Sir,
Your very humble Servant,
*To G. M. Burrows, Esq.* F. MILMAN.

---

No. IX. *Apothecaries Hall,*

SIR, *Dec.* 30, 1812.

I am desired by the Master and Wardens of the Society of Apothecaries, to acknowledge the receipt of your letter as Chairman of the Committee appointed by a General Meeting of the Apothecaries of England and Wales, on Friday the 20th of November last, which, together with the Report of the said Committee, were laid before a Court of Assistants on the 22d instant, when it was resolved, that its farther consideration should be deferred till they had obtained the opinion of the Royal College of Physicians on the same subject, which it appears by your letter has been submitted to them. I am, Sir,

Your obedient humble Servant,
S. BACKLER, C. K.

*Mr. G. M. Burrows, Chairman of the Committee appointed by a General Meeting of the Apothecaries of England and Wales, &c. &c. &c.*

---

No. X.
*Royal College of Surgeons,*
SIR, *Jan.* 18, 1813.

I have laid before the Court of Assistants your letter of the 11th, addressed to the Master, Governors, and Court of Assistants of this College, from the Committee appointed by a General Meeting of the Apothecaries of England and Wales, held at the Crown and Anchor Tavern, on Friday the 20th of November, 1812, together with a copy of the Report of the Committee, and the Resolutions of such General Meeting of the 20th of November; and am directed to inform you, that the Court of Assistants does not intend to interfere with the subject of such letter.

I am, Sir,
Your most obedient humble Servant,
*G. M. Burrows, Esq.* E. BELFOUR, Sec.

---

No. XI. *Bloomsbury-square,*

SIR, *Jan.* 21, 1813.

The Committee of Apothecaries having sketched a plan, which, upon mature deliberation, they consider adapted to the objects they have in view, respectfully beg leave to enclose a few copies of its outlines, which they hope you will take the trouble to lay before the other members of the Royal College of Physicians.

If the request with which they concluded their letter to the College of the 11th of December be assented to, the Committee will instantly submit the details of their plan for the consideration and approbation of yourself and the fellows, as it is their anxious desire to postpone their final arrangements until the last moment, to give all possible time for the decision of the College to be declared and received.

I have the honor to be, Sir,

Your obedient humble Servant,

*To Sir F. Milman, Bart.* G. M. Burrows, Chairman.

---

No. XII.

*Royal College of Physicians,*

Sir, *Jan.* 22, 1813.

I am directed to enclose you a copy of a Resolution passed at a meeting of the College this day; viz.

The College of Physicians cannot entertain the proposals from the London Committee, naming themselves the Committee of Apothecaries of England and Wales, for improving and protecting the profession of the Apothecary, Surgeon-Apothecary, and Practitioner in Midwifery, until they shall have received some official communications on the subject from the other chartered bodies, which are more immediately interested in the proposals.

I am, Sir,

Your obedient Servant,

*G. M. Burrows, Esq.* J. Hervey, Reg.

---

No. XIII.

The following Note was sent, with the outlines of the proposed plan, to the Royal College of Surgeons, and to the Society of Apothecaries:—

The Committee of Apothecaries beg leave to present to the
of the of copies of the outlines of their proposed plan for improving and protecting the profession of the apothecary, surgeon-apothecary, and practitioner of midwifery, in England and Wales.

*Holles-street, Jan.* 22, 1813. W. T. Ward, Sec.

---

No. XIV. *Bloomsbury-Square,*

Gentlemen, *Jan.* 27, 1813.

The answers of the Royal Colleges of Physicians and Surgeons

Surgeons to the letters of the Committee of Apothecaries of England and Wales, of Dec. 11th ultimo, were last night submitted to the Committee.

At the request of the Committee, I have the honor to enclose to the Master, Wardens, and Court of Assistants of the Society of Apothecaries, copies of those answers.

As the time for applying to parliament is so near as the 2d of February, the Committee take the liberty of soliciting, if there be no competent court held in the interim, that the master would have the goodness to summon a special court, to take the said answers of the Royal Colleges, and the application of the Committee, into consideration.

I have the honor to be, Sir,

Your obedient humble Servant,

G. M. Burrows, Chairman.

*To the Master, Wardens, and Court of Assistants of the Society of Apothecaries.*

---

No. XV. *Bloomsbury-square, Jan.* 28, 1813.

SIR,

I have laid before the Committee of Apothecaries the Resolution of the Royal College of Physicians of the 22d instant, in answer to their letter dated December 11, 1812, and communicated to me by the Registrar of the College on the 24th.

The Committee beg leave to present you with copies of the answers returned by the Royal College of Surgeons and the Society of Apothecaries to the aforesaid letter, which the Committee request you will do them the honor to lay before the Royal College of Physicians.

The Committee immediately submitted a copy of the resolution of the College of Physicians to the Master, Wardens, and Court of Assistants of the Society of Apothecaries.

The Committee respectfully suggest to the President and the Fellows of the Royal College of Physicians, that the two other chartered medical bodies, to whom the College of Physicians refer as more immediately interested in the proposals for improving and protecting the profession of the apothecary, surgeon-apothecary, and practitioner in midwifery, have in their answers most unequivocally acknowledged the body which has addressed the three chartered medical bodies as a Committee, elected at a meeting of the Apothecaries of England and Wales.

I have the honor to be, Sir,

Your obedient humble Servant,

*To Sir F. Milman, Bart.* G. M. Burrows, Chairman.

No. XVI.

SIR, *Brook-street, Jan.* 31, 1813.

I have received your letter of the 28th of January, inclosing copies of letters to you from the Royal College of Surgeons and from the Society of Apothecaries. I am bound by the resolution of the College of Physicians on the 22d of this month, a copy of which has by their command been sent to you, to wait for communications addressed to them by the corporate bodies above mentioned, before I can take any further steps in this business.

I have the honor to be, Sir,

Your most obedient humble Servant,

*To G. M. Burrows, Esq.* F. MILMAN.

---

No. XVII.

SIR, *Apothecaries Hall, Feb.* 5, 1813.

I am directed by the Master and Wardens of the Society of Apothecaries to inform you, that, in compliance with the request of your Committee, they have summoned a special Court of Assistants, to take into consideration the answers you have received from the Royal College of Physicians and Surgeons; and I am directed to send you their Resolution on that subject. I am, Sir,

Your obedient humble Servant,

*G. M. Burrows, Esq.* I. BACKLER, Cl.

*Apothecaries Hall, Feb.* 4, 1813.

At a special Court of Assistants held this instant:—

"Resolved—That this Court, having taken into consideration the reply of the Royal College of Physicians to a letter addressed to them from the Committee of Apothecaries of England and Wales, cannot (as a body) concur with that Committee in their intended application to parliament."

(A copy) I. BACKLER, Cl.

---

No. XVIII.

SIR, *Ely-place, Feb.* 18, 1813.

The College of Physicians having directed me to obtain a copy of the bill proposed to be brought into parliament by the Society of Apothecaries, I applied to my friend Mr. Wells, who has been so obliging as to furnish me with the outlines of the plan, and to refer me to you for a copy of the bill. I will, therefore, thank you to furnish me with a copy as soon as it is printed, or to favor me with the loan of the draft of it in the mean time, that I may lay it before the College for their consideration; it being the wish of the Society, as I am informed by Mr. Wells, to have the concurrence of the College in their proposed plan. I shall beg the favor to be informed when leave is given to bring in the bill, and the progress of it, &c. and am,

Sir, your obedient Servant,

JOHN ROBERTS.

*To Charles Druce*, Esq. Billiter-square.*

---

No. XIX. *Bloomsbury-square, Feb. 23, 1813.*

SIR,

Your letter of the 13th to Mr. Druce, requesting, at the desire of the College of Physicians, a copy of the bill prepared to be brought into parliament, has been sent to me; and I have, herewith, the pleasure of forwarding the printed abstract for that purpose. I lament it has been so long delayed, but it is to-day only that I could procure it from the printer's.

In presenting this document to you, to lay before the College, I cannot omit this or any other opportunity of expressing that the Committee of Apothecaries still entertain the same solicitude for the sanction and concurrence of the College, in the present application to parliament, on a measure so intimately connected with the public welfare. They will at all times be ready to manifest the sincerity of their intentions; and in any way that the College will condescend to point out.

Our solicitor, Mr. Druce, has instructions to communicate every information required by any of the constituted medical bodies. I am, Sir,

Your obedient humble Servant,

*To J. Roberts, Esq. Ely-place.* G. M. BURROWS.

Copies of the abstract were at the same time sent to the Royal Colleges of Physicians and Surgeons, and to Apothecaries Hall.

---

*To the Editor of the Medical and Physical Journal.*

SIR,

I ATTENDED some time ago, in my professional capacity as physician, a person laboring under diseased liver and dropsy, whose urine, while unusually thick and turbid, much abounded with bile.

---

* Solicitor for the Committee of Apothecaries and Surgeon-Apothecaries of England and Wales.

